# Acute Effects of Naturally Occurring Guayusa Tea and Nordic Lion’s Mane Extracts on Cognitive Performance

**DOI:** 10.3390/nu15245018

**Published:** 2023-12-06

**Authors:** Michael B. La Monica, Betsy Raub, Ethan J. Ziegenfuss, Shelley Hartshorn, Jodi Grdic, Ashley Gustat, Jennifer Sandrock, Tim N. Ziegenfuss

**Affiliations:** The Center for Applied Health Sciences, Canfield, OH 44406, USA

**Keywords:** cognitive function, mood, focus, concentration, nootropics, *Hericium erinaceus*

## Abstract

The aim of this study was to assess the effects of guayusa extract and Nordic Lion’s Mane (LM) on cognition. Using a randomized, double-blind, placebo-controlled, crossover design, we examined the effects of a single dose of 650 mg guayusa extract (AMT: AmaTea^®^ Max) vs. 1 g Nordic-grown Lion’s Mane (LM) vs. placebo (PL). Participants attended three testing visits consisting of neuropsychological tests (Go/No-go, N-Back, and Serial 7 s tasks) assessing performance, subjective assessments of cognitive perception, and vital signs. Each assessment was measured at baseline (pre-ingestion) and 1 and 2 h post ingestion. AMT significantly (*p* ≤ 0.05) improved the number of attempts during Serial 7s, total score, number of correct responses, total number of responses, and reaction time during N-Back and improved Go stimulus reaction time, but it reduced the percentage of correct responses in the No-go stimulus response during Go/No-go. LM significantly (*p* ≤ 0.05) improved the number of attempts during Serial 7s and reaction time during N-Back and improved Go stimulus reaction time in Go/No-go. AMT improved mental clarity, focus, concentration, mood, and productivity at 1 and 2 h (*p* < 0.05); the ability to tolerate stress at 1 h; and had greater ratings than LM and PL for mental clarity, focus, concentration, and productivity. PL improved focus and concentration at 1 h from baseline (*p* ≤ 0.05). AMT and LM improved subjective ratings of “happiness compared to peers” and “getting the most out of everything” (*p* < 0.05); however, this occurred earlier in LM (i.e., 1 h post ingestion). AMT uniquely elevated blood pressure from baseline. AMT significantly improved cognitive performance and self-perceived cognitive indices of affect over a 2 h period and perceptions of happiness 2 h post ingestion. In comparison, LM helped improve working memory, complex attention, and reaction time 2 h post ingestion and perceptions of happiness over a 2 h period.

## 1. Introduction

Guayusa (guayusa extract) is a naturally occurring tea currently available as a GRAS food ingredient. AmaTea^®^ Max (AMT) is produced from guayusa and includes methylxanthines (i.e., caffeine), chlorogenic acids, phenolic compounds, and terpenoids [1]. Guayusa leaves come from a tropical evergreen (*illex*) tree located in the upper Amazon basin of Ecuador, Columbia, and Peru and are noted for their stimulant and antioxidant properties [1,2]. Guayusa has had a long history of traditional use and has been deemed safe for consumption [1,3]. Caffeine is the most prevalent methylxanthine in guayusa [2], and since caffeine is known to enhance cognition and mood [4], guayusa extract would likely have a similar effect. Caffeine appears to exert its cognitive benefits through the blockade of adenosine receptors in dopamine-rich brain areas, thereby stimulating dopaminergic activity [5] and increasing neural activity (i.e., a reduction in alpha band frequency power, thus increasing cortical brain activity, alertness, and processing capabilities) [6]. Furthermore, chlorogenic acids, which are also highly abundant in guayusa, have been shown to offer enhancements in psychomotor speed and executive function [7]. The mechanism(s) by which chlorogenic acids exert cognitive improvements remain unclear, but Saitou et al. [7] showed an increase in serum concentration of cognitive impairment-linked biomarkers (i.e., apolipoprotein A1 (ApoA1) and Transthyretin (TTR)) levels after 16 weeks of daily chlorogenic acid supplementation. However, unlike caffeine, it is unclear if any cognitive enhancements from chlorogenic acids manifest acutely [7,8]. One recent study examined responses to acute (single dose) supplementation with guayusa (specifically AMT) on the acute impacts on cognitive function and e-gaming performance. Guayusa extract may have had a greater kills/match ratio vs. caffeine and placebo (*p* = 0.075), thereby potentially showing improvement in gaming performance (i.e., Fortnite) within a 4 h playing period [9]. Additionally, subjects reported that guayusa extract provided them with more vigor and reduced fatigue as compared to a placebo [9]. Interestingly, guayusa did not increase the sensation of jitters that have been commonly reported with caffeine [9]. Clearly, more studies are needed to examine the practical impact of AMT on cognition and affect.

The Nordic Lion’s Mane (*Hericium erinaceus*) extract utilized in this study is a GRAS ingredient (https://www.fda.gov/media/173805/download, accessed on 28 November 2023) extracted from an edible mushroom with a long history of use in Chinese medicine [10]. Lion’s Mane (LM) contains β-glucan polysaccharides, proteins, polyketides, hericenones and erinacine terpenoids, cyathane diterpenoids, lectins, phenols, isoindolinones, sterols, and myconutrients, which have been found to provide cognitive and neuroprotective properties [10,11]. *H. erinaceus* compounds may prevent and delay neurodegenerative diseases such as Alzheimer’s and Parkinson’s diseaseas [11,12,13]. Specifically, chemical compounds of *H. erinaceus* display pharmacological effects such as neurotrophic, antibiotic, anticarcinogenic, antidiabetic, antioxidant, immunomodulatory, and anti-neuroinflammatory activities [11,12,14,15]. The genomic and structural profile of *H. erinaceus* has been previously established [14,16]. 

The majority of studies on LM thus far have investigated its effects in regard to subjects with cognitive impairments and have shown benefits towards neurodegenerative diseases [17], while only a few have examined cognitive performance in healthy adults [18,19], and none have investigated the acute (i.e., single dose) cognitive effects. Erinacines within LM have demonstrated nootropic effects through the stimulation of nerve growth factor (NGF) synthesis, which plays a critical role in the growth, maintenance, and survival of neurons within the central nervous system [20], particularly the basal forebrain cholinergic system. 

In a rat model, erinacines have upregulated NGF levels in the hippocampus (where memory formation and spatial navigation take place) and the locus coeruleus (a small portion of the brainstem that plays a role in the regulation of arousal and attention) [21]. In addition, bioactive compounds from *H. erinaceus* have been able to stimulate NGF-mediated nerve outgrowth in rat neuronal cells [22]. In healthy humans over the age of 50 without cognitive decline, Saitsu et al. [18] observed improved recognition functions in an oral Mini-Mental State Examination over 12 weeks of LM (800 mg/day fruiting bodies) supplementation, whereas Grozier et al. [19] did not observe any improvements over 4 weeks (10 g/day of *H. erinaceus* in the form of a muffin) in cognitive flexibility with a Stroop test or in mathematical computations with a mental arithmetic task in a cohort of healthy college-aged participants. Thus, the need for acute examination of LM in healthy adults with normal cognition is warranted.

Given the dearth of scientific investigations examining both guayusa and LM on acute cognitive performance in healthy adults, this study aimed to assess the effects of AMT and LM on objective cognitive performance and subjective cognitive perception, including mental clarity, mood, focus, concentration, productivity, anxiety/stress, and happiness.

## 2. Methods

### 2.1. Experimental Design

This was a double-blind, randomized, three-arm, placebo-controlled, within-subject crossover trial in which participants visited the laboratory on four occasions (one screening visit and three testing visits). This study was conducted according to the guidelines laid down in the Declaration of Helsinki of 1975, and all procedures involving human subjects were approved by the Genetic Alliance IRB on 4/5/23 (#AFS-04-2023-003). Written informed consent was obtained from all subjects prior to enrollment. The study was registered on clinicaltrials.gov (#NCT06062186). This study was conducted at The Center for Applied Health Sciences, a contract research organization (CRO) in Northeast Ohio. During the initial screening visit, each participant’s medical history and blood work (CBC, CMP, and lipid panel) were assessed, baseline diet was evaluated, and each participant underwent 3 sets of familiarization trials of the neuropsychological assessments (Go/No-go, Serial Sevens, and N-Back). During the testing visits (visits 2–4), subjects completed baseline testing at 0 min (i.e., prior to acute supplementation), 60 min post ingestion, and 120 min post ingestion, which included subjective questionnaires [visual analog scales (VAS)] that assessed mood, focus, mental clarity, concentration, ability to be productive, ability to tolerate stress; a 4-item subjective happiness scale (SHS) that assessed general happiness, happiness in comparison to peers, enjoying life and getting the most out of everything, and level of unhappiness; and 3 sets of the neuropsychological assessments to assess reaction time, mental processing, cognitive control, and attention, and vital signs to assess blood pressure and heart rate. 

### 2.2. Participants

**Figure 1:**
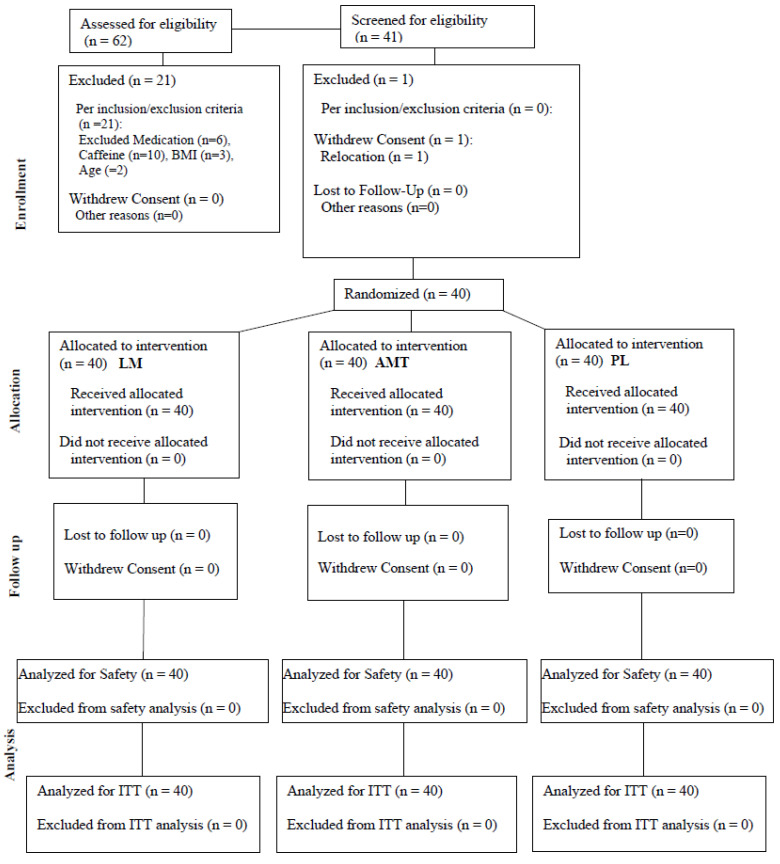
Consort diagram.

A total of 40 healthy men and women completed all study visits (see Table 1 for subject characteristics). All participants were in good health as determined by physical examination and medical history, between the ages of 18 and 50 years, had a body mass index (BMI) of 18.5–39.9 kg m^−2^, and habitually consumed ≤ 240 mg of caffeine per day. Prior to participation, all participants indicated their willingness to comply with all aspects of the experimental and supplement protocol. Participants were excluded if they (a) had a history of diabetes or pre-diabetes, hepatorenal, musculoskeletal, autoimmune, or neurologic disease; (b) had a history of malignancy in the previous 5 years except for non-melanoma skin cancer (basal cell cancer or squamous cell cancer of the skin); (c) had prior gastrointestinal bypass surgery; (d) had known gastrointestinal or metabolic diseases that might impact nutrient absorption or metabolism (e.g., short bowel syndrome, diarrheal illnesses, history of colon resection, gastroparesis, inborn errors of metabolism); (e) had any chronic inflammatory condition or disease; (f) had a history or current diagnosis of any mental disorders (i.e., cognitive or psychiatric); (g) were currently using thyroid, hyperlipidemic, hypoglycemic, anti-hypertensive, or anti-coagulant medication/s; (h) were currently pregnant or nursing; (i) were current smokers or nicotine users; (j) had a known allergy to any of the ingredients in the supplement or the placebo; (k) had recently participated in another research study with an investigational product or had been in another research study in the past 30 days; (l) used corticosteroids or testosterone replacement therapy (ingestion, injection, or transdermal); (m) had any other diseases or conditions that, in the opinion of the medical staff, could confound the primary endpoint or place the participant at increased risk of harm if they were to participate; or (n) did not demonstrate a verbal understanding of the informed consent document. See Figure 1 for a consort diagram.

Participants were instructed to follow their normal diet and activity patterns throughout their participation in the study. Participants were required to complete a 24 h diet record prior to arriving at the laboratory for their initial screening visit. Participants were provided a copy of this dietary record and instructed to duplicate all food intake 24 h prior to each subsequent laboratory visit. Prior to each subsequent visit, participants were asked to verbally confirm their 24 h prior diet adherence and ensure they had a normal night’s rest. In addition to replicating food intake for 24 h prior, study participants were also asked to refrain from exercise and alcohol 24 h prior, abstain from caffeine 12 h prior, and arrive 8 h fasted to all testing sessions, which were all verbally confirmed at the beginning of each study visit.

### 2.3. Neuropsychological Assessments

The Go/No-go task is designed to assess inhibitory responses. Stimuli were presented in a continuous stream, and participants were asked to perform a binary decision on each stimulus. Participants acted (i.e., pressed the spacebar on a keyboard) if the stimulus met the criteria (‘GO’ trial stimulus was presented as an orange square) or withheld their response (i.e., did not press the spacebar) if it did not meet the criteria (‘No-go’ trial stimulus was presented as a blue square). The task was designed to have more ‘GO’ (~81%) than ‘No-go’ stimuli (~19%), enticing the participant into a pattern of responses, thus making the ‘no-go’ trials more difficult in populations with poor impulse control. Accuracy and reaction time, two of our primary outcome measures, were recorded by the Testable stimulus presentation software (https://www.testable.org/experiment/12553/787858/start, accessed on 12 April 2023). The test was conducted for 90 s, and stimuli were presented once every 1000 ms with an additional 500 ms intertrial interval to allow for responses. Following an instructional slide, the task was presented in a block/square design such that 53 trials were presented. This task stimulates regions of the brain that are implicated in cognitive control and response inhibition [23].

The Serial Sevens test evaluates attention, concentration, and working memory and requires mathematical computations by subtracting seven from an initial starting value [24]. The initial starting value was set high enough so that the participant would not reach zero in the allotted time (i.e., 90 s). A random number between 294 and 300 or 494 and 500 was chosen by the researcher as an initial value (and as determined through familiarization testing). For example, if the starting value was 500, participants would correctly respond “493”, 486”, 479”, and so on as quickly as they could. If a participant provided an incorrect value, they were asked to try again; however, if the second attempt was incorrect, they were provided the correct response and asked to continue subtracting seven from that correct response. The total correct responses and incorrect responses were recorded for analysis. 

The N-Back task is widely used in the working memory literature [25]. It requires participants to determine if the current stimulus repeats relative to the item that occurred ‘n’ times before its onset. We used the 1-back task as a cognitive challenge; thus, this task would require participants to determine if the current stimulus is the same as the one before it. Stimuli were presented as a card with a colored shape for 1500 ms with a 500 ms intertrial interval. Participants had to respond as quickly as they could by striking an arrow on the keyboard corresponding to an appropriate response (e.g., either the color and shape matched, only the color or shape matched, or the color and shape did not match with the previous stimulus). The neural network actively involved in the N-Back includes an extensive frontoparietal network, encompassing regions involved in attention and decision making [25,26]. An overall score, accuracy (defined as the number of correct responses as a percentage and number of correct responses out of the total number of attempts), and reaction time, measured in ms, were recorded by the Luminosity stimulus presentation software (lumosity.com/app/v4/games/speed-match-overdrive-web, accessed 12 April 2023). Due to the limitations of the software, this test was performed back-to-back as two separate 45 s tests. The two tests were averaged to achieve one value for the score, accuracy (absolute and percentage), and reaction time per attempt.

Participants consecutively completed the Go/No-go task, Serial Sevens, and N-Back with one minute of rest in between each test and each set. In total, participants underwent 3 sets of Go/No-go tasks, Serial sevens, and N-Back at each time point, and the median value was taken as the value for each respective time point and used in the statistical analyses. During each testing visit, participants completed the Go/No-go task, Serial Sevens, and N-Back at baseline (before consumption of their respective supplement) and at 1 h, and 2 h post ingestion of their respective supplement.

### 2.4. Visual-Analog Scales

100 mm anchored VAS were completed before acute ingestion of the investigational supplement and 1 and 2 h after ingestion of each acute supplement on testing visits 2–4. VAS were anchored with “Worst possible” or “Lowest possible” and “Best possible” or “Highest possible” and assessed subjective ratings of mood, ability to focus, mental clarity, ability to concentrate, ability to be productive, and ability to tolerate stress. The validity and reliability of VAS to assess similar constructs have been previously established [27] and reported [28,29].

### 2.5. Subjective Happiness Scale (SHS)

The subjective happiness scale is a 4-item questionnaire that can be used to discern happy and unhappy tendencies. This assessment asked subjects to report how happy they feel in general, how happy they consider themselves compared to most of their peers, to what extent they enjoy life regardless of what is going on, getting the most out of everything, and how unhappy they are. The reliability and validity have been established previously [30].

### 2.6. Supplement Protocol

Throughout the study protocol, all ingredients were prepared in the form of two capsules for oral ingestion and packaged in coded generic containers for administration. Participants orally ingested a placebo (PL: maltodextrin), 650 mg guayusa extract (*Genus: Ilex*) (AMT: as AmaTea^®^ Max standardized at 20% caffeine (130 mg) and 30% chlorogenic acids), or 1 g 100% organic Nordic Lion’s Mane (*Hericium erinaceus*) fruiting bodies (LM). Using a Latin Square crossover design, participants were randomly assigned to receive the supplements in a specific balanced order such that approximately one-third received PL, another third received LM, and another third received AMT first. The supplement was consumed in the laboratory after their baseline testing timepoint in the presence of the research staff. Trials were no less than 48 h apart and no more than 7 days in between (i.e., in between visits 2, 3, and 4). All test products were verified for purity and potency by an independent third-party laboratory (Eurofins Scientific).

### 2.7. Anthropometric and Other Resting Measures

Standing height was determined using a wall-mounted stadiometer, and body weight (BW) was measured using a Seca 767TM Medical Scale (body weight was measured at each visit). Seated resting heart rate and blood pressure were measured using an automated blood pressure cuff (Omron HEM-780) at each timepoint (i.e., baseline, 1 h, and 2 h post after ingestion of each assigned supplement) during testing visits 2–4. 

### 2.8. Statistical Analyses

Primary outcome measures included cognitive performance based on the Serial Sevens test (number of errors made, number attempted), N-Back test (score, number of correct responses, number of attempted responses, accuracy (%), and average response time per attempt), and the Go/No-go Test (percent correct on Go, percent correct on No-go, reaction time on Go, reaction time on No-go). Secondary outcome measures included subjective changes in mental clarity, mood, focus, concentration, productivity, ability to tolerate stress, and subjective changes in happiness. Tertiary outcome measures included vital signs (blood pressure and heart rate), body weight, and side effect profile/adverse events monitoring. An a priori power analysis was conducted for the changes in VAS between treatments using G*Power. For a mixed factorial ANOVA with repeated measures, with three groups and three time points, within-between interaction, and a small effect of 0.25, a sample size of 36 was needed to achieve 80% power. All variables were tested for normality using results from a Shapiro–Wilk test. Data are presented as means ± standard deviation, and the primary statistical approach employed was mixed factorial ANOVA with repeated measures on time to assess group (AMT vs. LM vs. PL), time (baseline vs. 1 h vs. 2 h), and group x time interaction effects for all for cognitive performance testing, subjective changes in VAS and SHS indices, and vitals. A one-way ANOVA with repeated measures was completed to assess differences between conditions/groups for BW. When sphericity was violated, the Greenhouse–Geisser correction was used to adjust the p-values for main effects/interactions. Tukey post hoc procedures were used to assess individual comparisons between time points and/or groups. Main effects and interactions that indicated a significant difference (*p*-value of ≤0.05) or a trend (*p*-value of >0.5 to ≤0.1) were explored further, and post hoc outcomes were noted under the same conditions. Additionally, 95% confidence intervals (CIs) were calculated with each post hoc outcome, and mean changes from baseline were expressed as mean percent changes from baseline. Lastly, effect sizes using Cohen’s d (d) were calculated to evaluate the magnitude of the observed effect between conditions/groups (LM vs. AMT vs. PL) or time (baseline/0 min, 60 min, 120 min). All statistical analyses were conducted using GraphPad Prism version 10.

## 3. Results

A total of 22 women and 18 men completed all study visits. See Table 1.

### 3.1. Go/No-go

**Figure 2:**
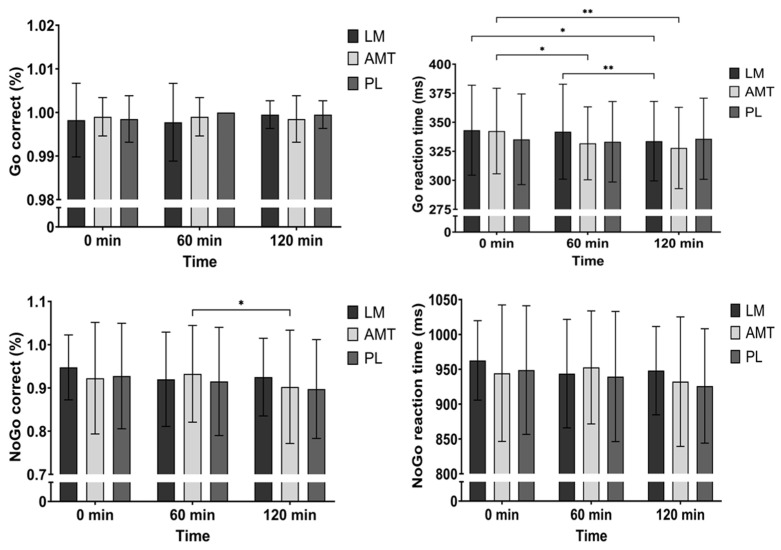
Go/No-go. * *p* ≤ 0.05; ** *p* ≤ 0.01. LM: Lion’s Mane. AMT: AmaTea. PL: placebo.

There were no differences between conditions over time for the percentage of correct Go stimuli (time: *p* = 0.231, group: *p* = 0.571, group × time: *p* = 0.271). However, there was a significant group × time interaction (*p* = 0.006) and a significant main effect of time (*p* = 0.008) for Go reaction time. Post hoc analysis between conditions showed that PL may have been slower than AMT at 120 min (mean difference −7.92 ms; *p* = 0.069, 95% CI: −16.3 to 0.50; d = 0.36). Meanwhile, post hoc analysis over time for LM indicated that reaction time was quicker at 120 min as compared to 0 min (2.6%, mean difference 9.43 ms; *p* = 0.036, 95% CI: 0.53 to 18.3; d = 0.41) and 60 min (2.3%, mean difference 8.18 ms; *p* = 0.004, 95% CI: 2.31 to 14.0; d = 0.54). Alternatively, post hoc analysis for AMT over time indicated that reaction time was quicker at 60 min (2.9%, mean difference 10.6 ms; *p* = 0.015, 95% CI: 1.80 to 19.3; d = 0.46) and 120 min (4.1%, mean difference 14.6 ms; *p* = 0.004, 95% CI: 4.21 to 24.9; d = 0.54) compared to 0 min. See Figure 2.

There was a time trend (*p* = 0.089) for the percentage of correct No-go stimuli. Furthermore, exploring the time effect, there was a worse percentage of correct No-go responses for AMT at 120 min as compared to 60 min (mean difference 3.0%; *p* = 0.023, 95% CI: 0. 35 to 5.65; d = 0.44). However, there were no differences between conditions over time for No-go reaction time (time: *p* = 0.105, group: *p* = 0.185, group × time: *p* = 0.346). See Figure 2.

### 3.2. Serial Sevens

**Figure 3:**
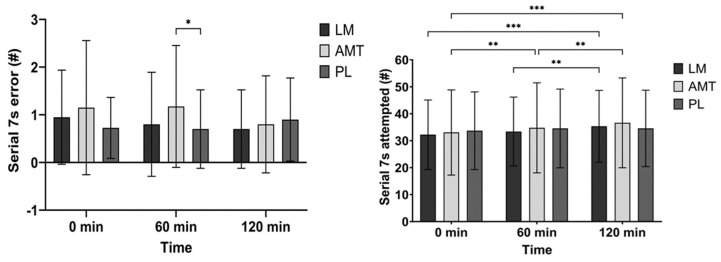
Serial Sevens. * *p* ≤ 0.05; ** *p* ≤ 0.01; *** *p* ≤ 0.001. LM: Lion’s Mane. AMT: AmaTea. PL: placebo.

There was a trend for an interaction (*p* = 0.084) for the number of incorrect responses during the Serial Sevens task. Post hoc analysis between conditions showed that AMT had more errors than PL at 60 min (71.4%, mean difference 0.48; *p* = 0.034, 95% CI: 0.03 to 0.92; d = 0.41). However, post hoc analysis over time for AMT indicated that errors may have improved at 120 min as compared to 0 min (33.3%, mean difference 0.35; *p* = 0.092, 95% CI: −0.05 to 0.75; d = 0.34) and 60 min (33.3%, mean difference 0.38; *p* = 0.059, 95% CI: −0.01 to 0.76; d = 0.37). See Figure 3.

### 3.3. N-Back

**Figure 4:**
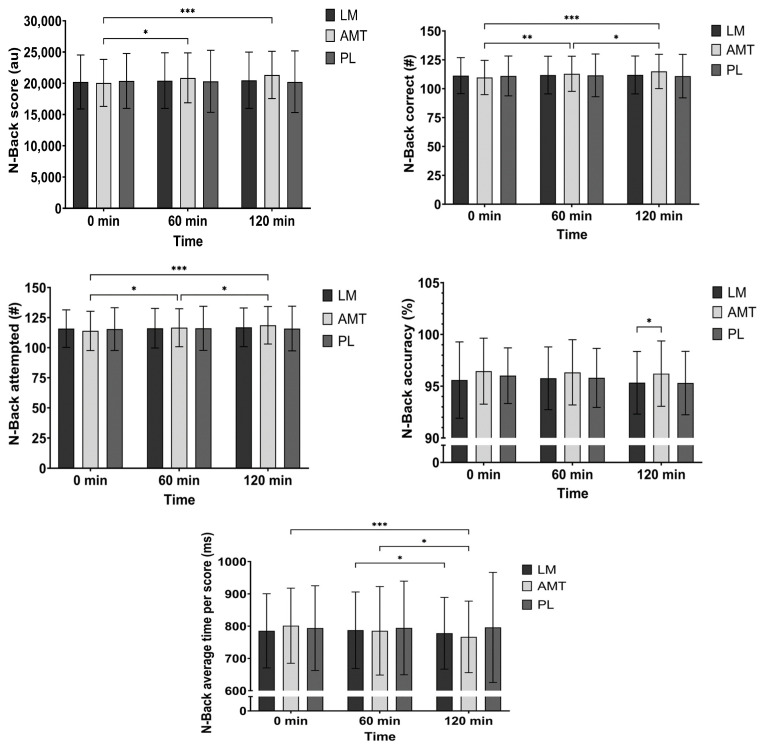
N-Back. * *p* ≤ 0.05; ** *p* ≤ 0.01; *** *p* ≤ 0.001. LM: Lion’s Mane. AMT: AmaTea. PL: Placebo.

There was a significant interaction (*p* = 0.011) and a significant main effect of time (*p* = 0.043) for N-Back score. Post hoc analysis for AMT over time indicated a higher score at 60 min (4.0%, mean difference −805 au; *p* = 0.018, 95% CI: −1489 to −121; d = 0.45) and 120 min (6.4%, mean difference −1279 au; *p* < 0.001, 95% CI: −1978 to −580; d = 0.71) as compared to 0 min and potentially a higher score at 120 min as compared to 60 min (2.3%, mean difference −474 au; *p* = 0.089, 95% CI: −1006 to 58.2; d = 0.34). See Figure 4.

There was a significant interaction (*p* = 0.002) and a significant main effect of time (*p* = 0.013) for the number of correct responses in the N-Back test. Post hoc analysis for AMT over time indicated more correct responses at 60 min (2.7%, mean difference −3.23; *p* = 0.005, 95% CI: −5.55 to −0.90; d = 0.53) and 120 min (4.5%, mean difference −5.23; *p* < 0.001, 95% CI: −7.48 to −2.97; d = 0.89) as compared to 0 min and more responses at 120 min as compared to 60 min (1.8%, mean difference −2.0; *p* = 0.017, 95% CI: −3.68 to −0.32; d = 0.46). See Figure 4.

There was a significant interaction (*p* = 0.009) and a significant main effect of time (*p* = 0.004) for the number of attempted responses in the N-Back test. Post hoc analysis for AMT over time indicated more correct responses at 60 min (2.2%, mean difference −2.65; *p* = 0.019, 95% CI: −4.92 to −0.38; d = 0.45) and 120 min (4.1%, mean difference −4.70; *p* < 0.001, 95% CI: −6.86 to −2.54; d = 0.84) as compared to 0 min and more responses at 120 min as compared to 60 min (1.8%, mean difference −2.05; *p* = 0.014, 95% CI: −3.73 to −0.37; d = 0.47). See Figure 4.

There was a main effect of condition (*p* = 0.034) and a time trend (*p* = 0.087) for N-Back accuracy. Further exploring the condition effect AMT indicated greater accuracy than LM (mean difference −0.89%; *p* = 0.017, 95% CI: −1.64 to −0.14; d = 0.46) and possibly PL (mean difference 0.91%; *p* = 0.077, 95% CI: −0.08 to 1.90; d = 0.35) at 120 min. See Figure 4.

There was a trend for an interaction (*p* = 0.074) and a time trend (*p* = 0.093) for N-Back time per score. Post hoc analysis over time for LM indicated that the time per score was quicker at 120 min as compared to 60 min (1.1%, mean difference 9.54 ms; *p* = 0.048, 95% CI: 0.06 to 19.0; d = 0.39). Alternatively, post hoc analysis over time for AMT indicated that the time per score was quicker at 120 min as compared to 0 min (4.2%, mean difference 34.5 ms; *p* < 0.001, 95% CI: 17.6 to 51.4; d = 0.79) and 60 min (2.2%, mean difference 18.6 ms; *p* = 0.038, 95% CI: 0.86 to 36.4; d = 0.40). See Figure 4. 

### 3.4. Visual Analog Scales (VAS)

**Figure 5:**
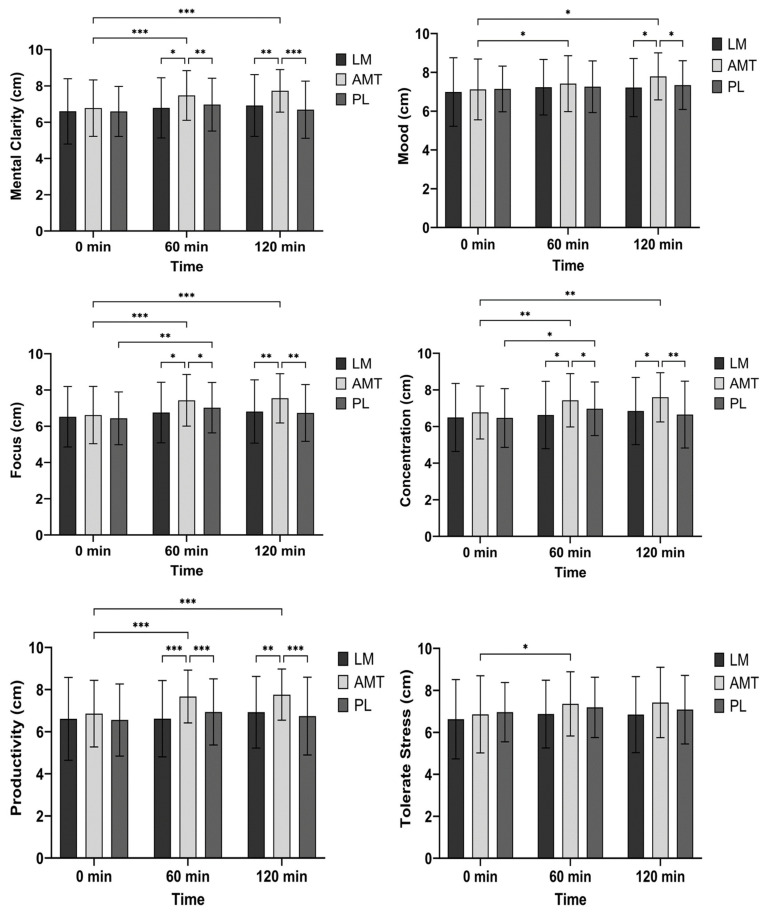
Visual analog scales. * *p* ≤ 0.05; ** *p* ≤ 0.01; *** *p* ≤ 0.001. LM: Lion’s Mane. AMT: AmaTea. PL: placebo.

There was a significant interaction (*p* = 0.013), a significant main effect of time (*p* = 0.001), and a significant main effect for condition (*p* = 0.002) for mental clarity. Post hoc analysis between conditions indicated that AMT had greater mental clarity than LM at 60 min (mean difference −0.68 cm; *p* = 0.017, 95% CI: −1.26 to −0.11; d = 0.46) and 120 min (mean difference −0.81 cm; *p* = 0.003, 95% CI: −1.37 to −0.25; d = 0.56) and PL at 60 min (mean difference −0.51 cm; *p* = 0.004, 95% CI: 0.15 to 0.87; d = 0.54) and 120 min (mean difference −1.04 cm; *p* < 0.001, 95% CI: 0.48 to 1.60; d = 0.72). Post hoc analysis over time indicated that AMT had greater mental clarity at 60 min (mean difference 8.18 cm; *p* < 0.001, 95% CI: 3.31 to 13.0; d = 0.68) and 120 min (mean difference 8.18 cm; *p* < 0.001, 95% CI: 3.31 to 13.0; d = 0.68) as compared to 0 min. See Figure 5.

There was a significant main effect of time (*p* = 0.014) for mood. Further exploring the time effect AMT improved mood at 60 min (mean difference −0.30 cm; *p* = 0.036, 95% CI: −0.57 to −0.02, d = 0.41) and 120 min (mean difference −0.67 cm; *p* = 0.010, 95% CI: −1.20 to −0.14; d = 0.49) as compared to 0 min and possibly improved mood at 120 min as compared to 60 min (mean difference −0.38 cm; *p* = 0.085, 95% CI: −0.79 to 0.04; d = 0.35). See Figure 5.

There was a trend for an interaction (*p* = 0.062), a significant main effect of time (*p* < 0.001), and a significant main effect of condition (*p* = 0.008) for focus. Post hoc analysis between conditions indicated that AMT had greater focus than LM at 60 min (mean difference −0.68 cm; *p* = 0.023, 95% CI: −1.27 to −0.08; d = 0.44) and 120 min (mean difference −0.73 cm; *p* = 0.008, 95% CI: −1.28 to −0.17; d = 0.50) and PL at 60 min (mean difference −0.41 cm; *p* = 0.041, 95% CI: 0.01 to 0.80; d = 0.40) and 120 min (mean difference 0.81 cm; *p* = 0.004, 95% CI: 0.23 to 1.39; d = 0.54). Post hoc analysis over time indicated that AMT had greater focus at 60 min (mean difference −0.82 cm; *p* < 0.001, 95% CI: −1.31 to −0.32, d = 0.63) and 120 min (mean difference −0.92 cm; *p* < 0.001, 95% CI: −1.50 to −0.35; d = 0.62) as compared to 0 min. Alternatively, PL had greater focus at 60 min as compared to 0 min (mean difference −0.59 cm; *p* = 0.006, 95% CI: −1.03 to −0.15, d = 0.52). See Figure 5.

There was a trend for an interaction (*p* = 0.090), a significant main effect of time (*p* < 0.001), and a significant main effect of condition (*p* = 0.005) for the ability to concentrate. Post hoc analysis between conditions indicated that AMT had a greater ability to concentrate than LM at 60 min (mean difference −0.80 cm; *p* = 0.029, 95% CI: −1.53 to −0.07; d = 0.42) and 120 min (mean difference −0.75 cm; *p* = 0.011, 95% CI: −1.35 to −0.15; d = 0.48) and PL at 60 min (mean difference −0.46 cm; *p* = 0.045, 95% CI: 0.01 to 0.92; d = 0.39) and 120 min (mean difference 0.94 cm; *p* = 0.005, 95% CI: 0.26 to 1.63; d = 0.53). Post hoc analysis over time indicated that AMT had a greater ability to concentrate at 60 min (mean difference −0.67 cm; *p* = 0.004, 95% CI: −1.14 to −0.20, d = 0.55) and 120 min (mean difference −0.83 cm; *p* = 0.001, 95% CI: −1.36 to −0.30; d = 0.60) as compared to 0 min. Alternatively, PL had a greater ability to concentrate at 60 min as compared to 0 min (mean difference −0.51 cm; *p* = 0.048, 95% CI: −1.01 to −0.00, d = 0.39). See Figure 5.

There was a significant interaction (*p* = 0.015), a significant main effect of time (*p* = 0.002), and a significant main effect for condition (*p* < 0.001) for productivity. Post hoc analysis between conditions indicated that AMT had greater productivity than LM at 60 min (mean difference −1.06 cm; *p* < 0.001, 95% CI: −1.70 to −0.41; d = 0.63) and 120 min (mean difference −0.84 cm; *p* = 0.001, 95% CI: −1.37 to −0.30; d = 0.60) and PL at 60 min (mean difference 0.73 cm; *p* < 0.001, 95% CI: 0.32 to 1.14; d = 0.69) and 120 min (mean difference 1.02 cm; *p* < 0.001, 95% CI: 0.41 to 1.63; d = 0.65). Post hoc analysis over time indicated that AMT improved productivity at 60 min (mean difference −0.82 cm; *p* < 0.001, 95% CI: −1.27 to −0.36, d = 0.69) and 120 min (mean difference −0.91 cm; *p* < 0.001, 95% CI: −1.45 to −0.37; d = 0.65) as compared to 0 min. Alternatively, LM possibly improved productivity at 120 min as compared to 60 min (mean difference −0.31 cm; *p* = 0.065, 95% CI: −0.63 to 0.02, d = 0.37) and PL possibly improved productivity at 60 min as compared to 0 min (mean difference −0.39 cm; *p* = 0.100, 95% CI: −0.83 to 0.06, d = 0.34). See Figure 5.

There was a significant main effect of time (*p* = 0.022) and a condition trend (*p* = 0.082) for the ability to tolerate stress. Further exploring the condition trend AMT possibly had a greater ability to tolerate stress than LM at 60 min (mean difference −0.49 cm; *p* = 0.082, 95% CI: −1.03 to 0.05; d = 0.35). Post hoc analysis over time indicated that AMT improved the ability to tolerate stress at 60 min as compared to 0 min (mean difference −0.50 cm; *p* = 0.020, 95% CI: −0.93 to −0.07, d = 0.44). See Figure 5.

### 3.5. Subjective Happiness Scale (SHS)

**Figure 6:**
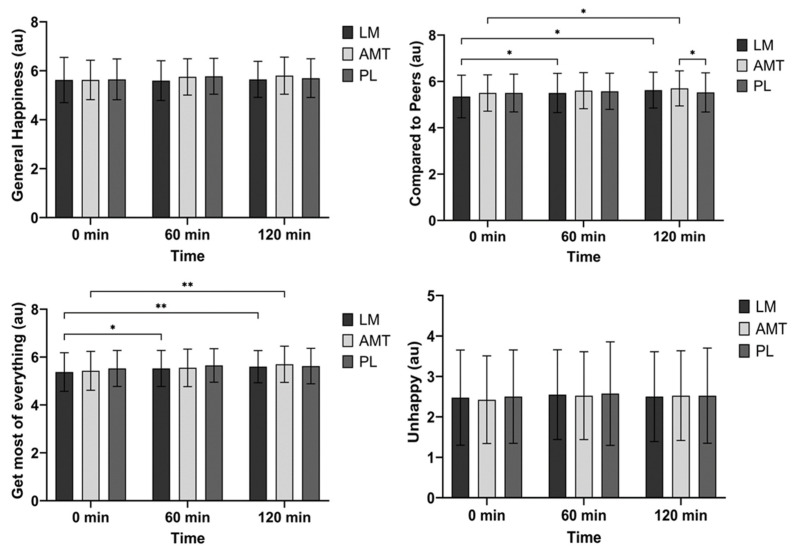
Subjective happiness scale. * *p* ≤ 0.05; ** *p* ≤ 0.01. LM: Lion’s Mane. AMT: AmaTea. PL: placebo.

There were no differences between groups over time for general happiness (time: *p* = 0.117, group: *p* = 0.334, group × time: *p* = 0.136). See Figure 6.

There was a significant main effect of time (*p* < 0.001) for happiness compared to peers. Post hoc analysis over time indicated that LM improved happiness compared to peers at 60 min (mean difference −0.15 au; *p* = 0.032, 95% CI: −0.29 to −0.01, d = 0.41) and 120 min (mean difference −0.28 au; *p* = 0.016, 95% CI: −0.51 to −0.04; d = 0.46) as compared to 0 min. Alternatively, AMT improved happiness compared to peers at 120 min as compared to 0 min (mean difference −0.20 au; *p* = 0.045, 95% CI: −0.38 to −0.02; d = 0.43). See Figure 6.

There was a significant main effect of time (*p* < 0.001) for getting the most out of everything. Post hoc analysis over time indicated that LM improved getting the most out of everything at 60 min (mean difference −0.15 au; *p* = 0.032, 95% CI: −0.29 to −0.01, d = 0.41) and 120 min (mean difference −0.23 au; *p* = 0.005, 95% CI: −0.39 to −0.06; d = 0.53) as compared to 0 min. Alternatively, AMT improved getting the most out of everything at 120 min as compared to 0 min (mean difference −0.28 au; *p* = 0.009, 95% CI: −0.49 to −0.06; d = 0.50) and possibly 60 min (mean difference −0.15 au; *p* = 0.080, 95% CI: −0.31 to 0.01; d = 0.35). Also, PL possibly improved getting the most out of everything at 60 min as compared to 0 min (mean difference −0.13 au; *p* = 0.059, 95% CI: −0.25 to 0.00; d = 0.37). See Figure 6.

There was a time trend (*p* = 0.078) for unhappiness; however, there were no significant post hoc differences. See Figure 6.

### 3.6. Hemodynamics, Body Weight, and Adverse Events

**Figure 7:**
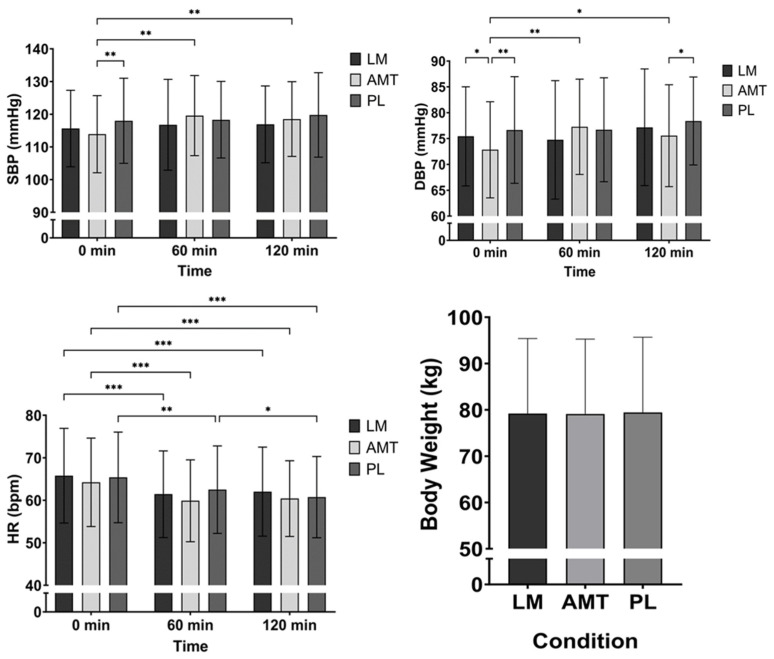
Hemodynamics and body weight. * *p* ≤ 0.05; ** *p* ≤ 0.01; *** *p* ≤ 0.001. LM: Lion’s Mane. AMT: AmaTea. PL: placebo.

There was a trend for an interaction (*p* = 0.094) and a significant main effect for time (*p* = 0.007) for SBP. Post hoc analysis between conditions indicated that PL had a higher SBP than AMT at 0 min (mean difference −4.1 mmHg; *p* = 0.010, 95% CI: −7.31 to −0.89; d = 0.49). Post hoc analysis over time for AMT indicated that SBP was higher at 60 min (mean difference −5.7 mmHg; *p* = 0.006, 95% CI: −9.84 to −1.51, d = 0.52) and 120 min (mean difference −4.6 mmHg; *p* = 0.006, 95% CI: −8.05 to −1.20, d = 0.52) as compared to 0 min. See Figure 7.

There was a significant interaction (*p* = 0.002), a significant main effect for time (*p* = 0.009), and a condition trend (*p* = 0.080) for DBP. Post hoc analysis between conditions indicated that AMT had a lower DBP than LM at 0 min (mean difference 2.6 mmHg; *p* = 0.046, 95% CI: 0.042 to 5.16; d = 0.39) and PL at 0 min (mean difference −3.8 mmHg; *p* = 0.005, 95% CI: −6.59 to −1.06; d = 0.53) and 120 min (mean difference −2.9 mmHg; *p* = 0.026, 95% CI: −5.40 to −0.30; d = 0.43). Post hoc analysis over time for AMT indicated that DBP was higher at 60 min (mean difference −4.5 mmHg; *p* = 0.001, 95% CI: −7.23 to −1.67, d = 0.62) and 120 min (mean difference −2.7 mmHg; *p* = 0.021, 95% CI: −5.09 to −0.36, d = 0.44) as compared to 0 min and LM was possibly higher at 120 min as compared to 60 min (mean difference −2.4 mmHg; *p* = 0.067, 95% CI: −5.0 to 0.14, d = 0.36). See Figure 7.

There was a significant main effect for time (*p* < 0.001) for HR. Post hoc analysis over time indicated a lower HR at 60 min and 120 min as compared to 0 min for LM (60 min: mean difference 4.4 bpm; *p* < 0.001, 95% CI: 2.17 to 6.53, d = 0.77; 120 min: mean difference 3.8 bpm; *p* < 0.001, 95% CI: 1.54 to 5.96, d = 0.65), AMT (60 min: mean difference 4.4 bpm; *p* < 0.001, 95% CI: 2.40 to 6.30, d = 0.86, 120 min: mean difference 3.8 bpm; *p* < 0.001, 95% CI: 1.63 to 6.02, d = 0.67), and PL (60 min: mean difference 2.9 bpm; *p* = 0.004, 95% CI: 0.86 to 4.89, d = 0.55, 120 min: mean difference 4.6 bpm; *p* < 0.001, 95% CI: 2.71 to 6.54, d = 0.93). In addition, PL had a lower HR at 120 min as compared to 60 min (mean difference 1.8 bpm; *p* = 0.019, 95% CI: 0.25 to 3.26, d = 0.45). See Figure 7.

There was a time trend (*p* = 0.069) for body weight, indicating that participants possibly had greater body weight during PL than AMT (mean difference −0.3 kg; *p* = 0.069, 95% CI: −0.64 to 0.02; d = 0.36). See Figure 7.

Three out of forty participants suffered mild adverse events. During the AMT trial, two participants experienced either a dry mouth or tremors, while during the PL trial, one participant experienced a headache. No AEs were reported during the LM trial.

## 4. Discussion

This acute, crossover, placebo-controlled investigation sought to assess the effectiveness of guayusa extract in the form of AMT and LM (*Hericium erinaceus*) on cognitive performance and subjective feelings of cognition and happiness. Indeed, broad, significant cognitive improvements were seen in AMT (i.e., number of correct and attempted responses, reaction time, and total score) at 1 and 2 h post ingestion across all neuropsychological assessments, while LM had some improvements (i.e., number of attempted responses and reaction time) that were observed at 2 h post ingestion. However, compared to PL, AMT had a greater number of errors in the Serial Sevens test at 1 h post ingestion. Likewise, all self-reported cognitive indices were improved in AMT at 1 and 2 h post ingestion, while under the PL condition, participants only improved concentration and focus at 1 h post ingestion. Compared to PL and LM, AMT reported greater feelings of mental clarity, focus, concentration, and productivity at 1 and 2 h post ingestion. Both LM and AMT positively influenced subjective ratings of happiness, with LM exerting its influence earlier than AMT over the 2 h testing period. Blood pressure was uniquely elevated up to 5–6 mmHg in SBP and 3–4 mmHg in DBP, on average, for 2 h post ingestion in AMT. Lastly, all conditions were well tolerated, with few adverse events.

### 4.1. Cognitive Performance

In this study, we selected participants who were habitual caffeine consumers of low to moderate intake (<240 mg/day). We observed marked improvements in executive function within the N-back test for AMT. Specifically, AMT significantly improved the total score (a combination of consistent accuracy and speed) in the first hour and possibly (*p* = 0.089) again in the second hour, and improved the number of correct responses, the total number of responses, and reaction time in the first hour and again in the second hour. Although AMT did not improve accuracy over time, it was more accurate than LM (and possibly PL (*p* = 0.077)) at 2 h post ingestion. LM improved reaction time from 60 min to 120 min, potentially showing a delayed response after ingestion. During the Serial Sevens test, AMT improved the number of attempted responses at the first hour and again at the second hour, while LM improved the number of attempted responses after 2 h (as compared to baseline and 60 min, showing a potential delayed response). AMT may have also reduced the number of errors in the Serial Sevens test made in the second hour from baseline (*p* = 0.092) and possibly from the first hour (*p* = 0.059). 

During the Go/No-go test, AMT improved reaction time from baseline to the stimulatory response (i.e., GO) in the first hour and maintained it during the second hour. Meanwhile, LM also improved reaction time to the stimulatory response at 120 min (from baseline and 60 min), again suggesting a delayed response to the ingestion of the supplement. Unlike other benefits seen in AMT, there was a reduction (i.e., worse) in the number of correct responses to the inhibitory stimuli (i.e., No-go correct) from 60 to 120 min as well as potentially lowering the reaction time (i.e., participants responded quicker to the inhibitory stimuli, which is also worse) from 60 to 120 min. This may have occurred because AMT contains caffeine, which may negatively affect an individual’s impulse control on inhibiting actions [5]. Thus, there may have been enough relative caffeine in AMT to cause a sufficient arousal in participants, who were not naturally impulsive, causing them to lack control of their actions. This may especially hold true since all the neuropsychological tests in this study instructed the participants to act and respond as quickly as possible. Therefore, the small percentage of No-go trials within the Go/No-go task (and within all the neuropsychological tests) were experienced as a novel challenge to the participants as it countered the instructions that were administered in all other cognitive tasks within the testing battery.

In this experimental design, caffeine is likely to be the most profound contributor to the observed cognitive performance in AMT, particularly in the N-back test where cognitive domains such as decision making, selection, working memory, suppression, and interference separation processes are incorporated [26], all of which can be improved by caffeine [4,5,6,31]. Caffeine may also have a positive influence on basic psychomotor tasks [5] and preparatory attention [6] as needed in the Go/No-go challenge. It has been shown that caffeine increases alertness via reductions in alpha band frequency power, reflecting an increase in cortical brain activity, thereby resulting in enhanced selective attention and sensitivity to visual information [6]. Similar to the cognitive demands in the current investigation, but with different neuropsychological tasks, Haskell et al. [32] observed improvements in simple choice reaction time, digit vigilance reaction time, numeric working memory reaction time, and accuracy in a sentence verification task among habitual caffeine consumers. Additionally, Brunye et al. [5] found that moderate and high levels of caffeine improved participants’ ability to take advantage of alerting cues and inhibit their action toward incompatible information (slightly contradictory to what we observed), leading to positive influences on basic psychomotor tasks and demonstrating that caffeine can aid performance on tasks requiring sustained attention and vigilance. In contrast to the current finding, Bloomer et al. [9] did not observe any significant differences between AMT, an equal dose of caffeine, or PL in the Go/No-go test, digit symbol substitution test, or a 30 min mental performance test. However, the difference in results between Bloomer et al. [9] and our investigation is possibly due to the much longer and more mentally grueling battery of tests within Bloomer and colleagues’ study, as the testing session was approximately 6 h with a 4 h e-gaming session, whereas participants in the current investigation were tested for approximately 25 min, three times, within a 3 h period. Also, Bloomer and colleagues tested subjects during the evening hours as compared to the morning hours, as was the case in the current investigation. Furthermore, their subjects arrived 3 h fasted and consumed 270 mg of caffeine within AMT vs. an 8 h fast and 130 mg of caffeine within the current investigation.

In contrast to AMT, the nootropic benefits of LM appear to be due to the presence of hericinones and L-ergothioneine [17]. However, it is important to note that much of the literature supporting the nootropic benefits of LM has been carried out in rodent models (i.e., rats and mice). Only a few studies have examined the cognitive adaptations to LM in the elderly with normal cognition [18], college-aged cohort [19], and a pilot study in the elderly with cognitive impairment [33]. In particular, erinacine A can stimulate NGF synthesis, promote NGF-induced neurite outgrowth stimulation, and protect against the decline or death of neurons in the brain, as shown in a rat model [21]. Erinacine A has been shown to upregulate NGF levels in parts of the brain responsible for memory formation, arousal, and attention (i.e., hippocampus and locus coeruleus) within a rat model [21]. Also, after oral consumption in a rat model, Tsai et al. [34] observed erinacine A was present in the brain one hour after ingestion, suggesting that passive diffusion through the blood–brain barrier was the dominant transport method [17]. Since the fruiting body does not contain quantifiable levels of erinacines, it can be suggested that during metabolism, the hericenone metabolites are converted to erinacines, or they, too, have the ability to impact NGF or a different mechanism of action altogether [12,32,33,34]. In mice, ergothioneine has been shown to reduce oxidative stress and inflammation in the cerebellum and hippocampus and increase neurotransmission of glutamate, an amino acid and neurotransmitter responsible for communication between neurons in the brain, owing to its nootropic benefit such as an attenuation in recognition memory [35]. 

### 4.2. Visual Analog Scales (VAS) and Subjective Happiness Scale (SHS)

AMT significantly improved mental clarity, focus, concentration, mood, and productivity at 60 and 120 min post ingestion, and the ability to tolerate stress at 60 min while also having significantly greater cognitive responses than LM and PL at 60 and 120 min post ingestion for mental clarity, focus, concentration, productivity, and perhaps mood (*p* < 0.10). Alternatively, PL improved focus, concentration, and perhaps productivity at 60 min from baseline, likely the result of the well-documented “placebo effect” that is especially prevalent in subjective outcomes [36]. On the other hand, caffeine, in AMT, blocks adenosine activity as an A2a receptor antagonist, which may help maintain focus and mental energy by stimulating the central nervous system [31]. Haskell et al. [32] reported reduced mental fatigue, tiredness, and increased alertness after 75 mg and 150 mg of caffeine in habitual consumers of caffeine. Meanwhile, Bloomer et al. [9] reported no differences in attentiveness, energy, motivation, irritability, or mood but observed a statistical trend (*p* = 0.07) in focus, suggesting higher levels under the AMT condition over a 4 h period. Additionally, Bloomer et al. [9] noticed a conditional effect for fatigue (showing AMT had lower levels than PL) and for vigor (higher values in AMT vs. PL). However, in Bloomer et al. [9], the caffeine-matched group also had minimal effects on subjective ratings. Since caffeine has often been associated with greater feelings of anxiety/jitteriness, we measured the ability to tolerate stress, in which a lower score would have indicated a reduced ability to handle stress/anxiety. Remarkably, AMT observed a higher score, indicating a greater potential to handle stress. The reason for this may been due to the other bioactive compounds within AMT [2], such as epigallocatechin gallate (EGCG) [37] and theanine [31], which have been shown to increase subjective calmness and reduce stress, and this observation coincides with the previous literature on AMT showing no effects on subjective jitteriness [9].

LM significantly improved subjective ratings of happiness compared to peers and “getting the most out of everything” at 60 min (from baseline) and maintained it at 120 min, whereas AMT significantly improved both of those ratings only at 120 min from baseline. However, AMT did have a significantly better rating of ‘happiness compared to peers’ than PL at 120 min post ingestion. On the other hand, PL may have improved the subjective rating of getting the most out of everything at 60 min from baseline. Overall, it appeared that LM had an influence on the happiness scale at 60 min, whereas AMT’s observances came to fruition at 120 min. Studies on LM’s impact on mood are limited, especially acute designs such as the current investigation, but one pilot study showed that *H. erinaceus* decreased depression, anxiety, and sleep disorders with an associated elevation in peripheral levels of pro-BDNF over an 8-week treatment period [38]. This may suggest how LM could alter mood states or act as an antidepressant and subsequently increase happiness over a longer period of use, but the acute setting is still unclear. In theory, LM is known to ameliorate gastrointestinal diseases and potentially modulate the gut microbiota, which influences the gut–brain axis and, in turn, could influence mood states and/or depression [17,39,40]. 

### 4.3. Safety and Vitals

AMT uniquely and significantly elevated SBP (6 and 5 mm Hg on average) and DBP (4 and 3 mm Hg on average) at 60 min and maintained higher values at 120 min, respectively, from baseline. On the other hand, HR in all conditions was significantly reduced by 60 min and maintained at 120 min (LM and AMT) or even further reduced at 120 min (PL) from baseline. In contrast, Bloomer et al. [9] did not find any differences in DBP or HR between AMT, caffeine, and PL, but the authors did observe a lower SBP in the PL condition than caffeine and AMT. Acute ingestion of caffeine has been reported to raise blood pressure in healthy individuals; however, results can be contradictory depending on the habituation status of the individual(s) [41]. Despite caffeine’s potential for an acute transient increase in blood pressure, it is important to note that the average blood pressure values observed in this study were well within normotensive values. Lastly, all conditions were well tolerated, with minimal adverse events reported (3 out of a possible 120), which were considered mild in nature.

## 5. Conclusions

This unique investigation measured the acute benefits of AMT and LM on objective cognitive performance, subjective cognitive perception, and self-perceived happiness in low to moderate caffeine consumers. To our knowledge, this is the first study to examine the effects of LM on cognitive performance in healthy subjects. AMT significantly improved cognitive performance and self-perceived cognitive indices of affect over a 2 h period and perceptions of happiness 2 h post ingestion. In comparison, LM helped improve working memory, complex attention, and reaction time 2 h post ingestion and perceptions of happiness over a 2 h period. Although this study shows that continuous intake of LM is not necessary to stimulate positive cognitive benefits, a longer duration or higher doses of LM use may be needed to remodel nerve structures in the brain and potentially provide even greater effects acutely and/or chronically. In addition, the antioxidant effects of LM [17] and AMT [2,42] may provide additional health benefits or more consistent improvements in cognition with chronic use. Future studies should utilize EEG and/or fMRI data to provide more insight into the cognitive improvements we observed.

## Figures and Tables

**Table 1:** Participant characteristics.

Age (years)	34.0 ± 9.5
Height (cm)	171.8 ± 11.8
Weight (kg)	79.1 ± 16.2
Body Mass Index (kg/m^2^)	26.6 ± 3.6
Systolic Blood Pressure (mm Hg)	118.3 ± 11.7
Diastolic Blood Pressure (mm Hg)	75.6 ± 8.5
Resting Heart Rate (bpm)	67.1 ± 12.0

## Data Availability

Restrictions apply to the availability of these data. The data that support the findings of this study are available by reasonable request from the authors upon permission from the sponsor (Applied Food Sciences Inc.).

## References

[1] Wise G., Negrin A. (2020). A Critical Review of the Composition and History of Safe Use of Guayusa: A Stimulant and Antioxidant Novel Food. Crit. Rev. Food Sci. Nutr..

[2] Kapp R.W., Mendes O., Roy S., McQuate R.S., Kraska R. (2016). General and Genetic Toxicology of Guayusa Concentrate (Ilex Guayusa). Int. J. Toxicol..

[3] Krieger D., Kalman D., Feldman S., Arnillas L., Goldberg D., Gisbert O., Nader S. (2016). The Safety, Pharmacokinetics, and Nervous System Effects of Two Natural Sources of Caffeine in Healthy Adult Males. Clin. Transl. Sci..

[4] Lorenzo Calvo J., Fei X., Domínguez R., Pareja-Galeano H. (2021). Caffeine and Cognitive Functions in Sports: A Systematic Review and Meta-Analysis. Nutrients.

[5] Brunyé T.T., Mahoney C.R., Lieberman H.R., Taylor H.A. (2010). Caffeine Modulates Attention Network Function. Brain Cogn..

[6] van den Berg B., de Jong M., Woldorff M.G., Lorist M.M. (2021). Caffeine Boosts Preparatory Attention for Reward-Related Stimulus Information. J. Cogn. Neurosci..

[7] Saitou K., Ochiai R., Kozuma K., Sato H., Koikeda T., Osaki N., Katsuragi Y. (2018). Effect of Chlorogenic Acids on Cognitive Function: A Randomized, Double-Blind, Placebo-Controlled Trial. Nutrients.

[8] Camfield D.A., Silber B.Y., Scholey A.B., Nolidin K., Goh A., Stough C. (2013). A Randomised Placebo-Controlled Trial to Differentiate the Acute Cognitive and Mood Effects of Chlorogenic Acid from Decaffeinated Coffee. PLoS ONE.

[9] Bloomer R.J., Martin K.R., Pence J.C. (2022). Impact of AmaTea^®^ Max on Physiological Measures and Gaming Performance in Active Gamers: A Placebo-Controlled, Double-Blind, Randomized Study. J. Clin. Transl. Res..

[10] Spelman K., Sutherland E., Bagade A. (2017). Neurological Activity of Lion’s Mane (*Hericium Erinaceus*). J. Restor. Med..

[11] Friedman M. (2015). Chemistry, Nutrition, and Health-Promoting Properties of *Hericium Erinaceus* (Lion’s Mane) Mushroom Fruiting Bodies and Mycelia and Their Bioactive Compounds. J. Agric. Food Chem..

[12] Wei J., Li J., Feng X., Zhang Y., Hu X., Hui H., Xue X., Qi J. (2023). Unprecedented Neoverrucosane and Cyathane Diterpenoids with Anti-Neuroinflammatory Activity from Cultures of the Culinary-Medicinal Mushroom *Hericium Erinaceus*. Molecules.

[13] Tzeng T.-T., Chen C.-C., Chen C.-C., Tsay H.-J., Lee L.-Y., Chen W.-P., Shen C.-C., Shiao Y.-J. (2018). The Cyanthin Diterpenoid and Sesterterpene Constituents of *Hericium Erinaceus* Mycelium Ameliorate Alzheimer’s Disease-Related Pathologies in APP/PS1 Transgenic Mice. Int. J. Mol. Sci..

[14] Wei J., Cheng M., Zhu J., Zhang Y., Cui K., Wang X., Qi J. (2023). Comparative Genomic Analysis and Metabolic Potential Profiling of a Novel Culinary-Medicinal Mushroom, *Hericium Rajendrae* (Basidiomycota). J. Fungi.

[15] Wu F., Zhou C., Zhou D., Ou S., Zhang X., Huang H. (2018). Structure Characterization of a Novel Polysaccharide from *Hericium Erinaceus* Fruiting Bodies and Its Immunomodulatory Activities. Food Funct..

[16] Gong W., Wang Y., Xie C., Zhou Y., Zhu Z., Peng Y. (2020). Whole Genome Sequence of an Edible and Medicinal Mushroom, *Hericium Erinaceus* (Basidiomycota, Fungi). Genomics.

[17] Brandalise F., Roda E., Ratto D., Goppa L., Gargano M.L., Cirlincione F., Priori E.C., Venuti M.T., Pastorelli E., Savino E. (2023). *Hericium Erinaceus* in Neurodegenerative Diseases: From Bench to Bedside and Beyond, How Far from the Shoreline?. J. Fungi.

[18] Saitsu Y., Nishide A., Kikushima K., Shimizu K., Ohnuki K. (2019). Improvement of Cognitive Functions by Oral Intake of *Hericium Erinaceus*. Biomed. Res..

[19] Grozier C.D., Alves V.A., Killen L.G., Simpson J.D., O’neal E.K., Waldman H.S. (2022). Four Weeks of *Hericium Erinaceus* Supplementation Does Not Impact Markers of Metabolic Flexibility or Cognition. Int. J. Exerc. Sci..

[20] Zhang C.-C., Cao C.-Y., Kubo M., Harada K., Yan X.-T., Fukuyama Y., Gao J.-M. (2017). Chemical Constituents from *Hericium Erinaceus* Promote Neuronal Survival and Potentiate Neurite Outgrowth via the TrkA/Erk1/2 Pathway. Int. J. Mol. Sci..

[21] Shimbo M., Kawagishi H., Yokogoshi H. (2005). Erinacine A Increases Catecholamine and Nerve Growth Factor Content in the Central Nervous System of Rats. Nutr. Res..

[22] Phan C.-W., Lee G.-S., Hong S.-L., Wong Y.-T., Brkljača R., Urban S., Malek S.N.A., Sabaratnam V. (2014). Hericium Erinaceus (Bull.: Fr) Pers. Cultivated under Tropical Conditions: Isolation of Hericenones and Demonstration of NGF-Mediated Neurite Outgrowth in PC12 Cells via MEK/ERK and PI3K-Akt Signaling Pathways. Food Funct..

[23] Swick D., Ashley V., Turken U. (2011). Are the Neural Correlates of Stopping and Not Going Identical? Quantitative Meta-Analysis of Two Response Inhibition Tasks. NeuroImage.

[24] Bristow T., Jih C.-S., Slabich A., Gunn J. (2016). Standardization and Adult Norms for the Sequential Subtracting Tasks of Serial 3’s and 7’s. Appl. Neuropsychol. Adult.

[25] Wang H., He W., Wu J., Zhang J., Jin Z., Li L. (2019). A Coordinate-Based Meta-Analysis of the n-Back Working Memory Paradigm Using Activation Likelihood Estimation. Brain Cogn..

[26] Jonides J., Schumacher E.H., Smith E.E., Lauber E.J., Awh E., Minoshima S., Koeppe R.A. (1997). Verbal Working Memory Load Affects Regional Brain Activation as Measured by PET. J. Cogn. Neurosci..

[27] Lee K.A., Hicks G., Nino-Murcia G. (1991). Validity and Reliability of a Scale to Assess Fatigue. Psychiatry Res..

[28] Lopez H.L., Cesareo K.R., Raub B., Kedia A.W., Sandrock J.E., Kerksick C.M., Ziegenfuss T.N. (2020). Effects of Hemp Extract on Markers of Wellness, Stress Resilience, Recovery and Clinical Biomarkers of Safety in Overweight, But Otherwise Healthy Subjects. J. Diet. Suppl..

[29] Ziegenfuss T.N., Kedia A.W., Sandrock J.E., Raub B.J., Kerksick C.M., Lopez H.L. (2018). Effects of an Aqueous Extract of Withania Somnifera on Strength Training Adaptations and Recovery: The STAR Trial. Nutrients.

[30] Lyubomirsky S., Lepper H.S. (1999). A Measure of Subjective Happiness: Preliminary Reliability and Construct Validation. Soc. Indic. Res..

[31] Kennedy D.O., Wightman E.L. (2022). Mental Performance and Sport: Caffeine and Co-Consumed Bioactive Ingredients. Sports Med..

[32] Haskell C.F., Kennedy D.O., Wesnes K.A., Scholey A.B. (2005). Cognitive and Mood Improvements of Caffeine in Habitual Consumers and Habitual Non-Consumers of Caffeine. Psychopharmacology.

[33] Li I.-C., Chang H.-H., Lin C.-H., Chen W.-P., Lu T.-H., Lee L.-Y., Chen Y.-W., Chen Y.-P., Chen C.-C., Lin D.P.-C. (2020). Prevention of Early Alzheimer’s Disease by Erinacine A-Enriched *Hericium Erinaceus* Mycelia Pilot Double-Blind Placebo-Controlled Study. Front. Aging Neurosci..

[34] Tsai P.-C., Wu Y.-K., Hu J.-H., Li I.-C., Lin T.-W., Chen C.-C., Kuo C.-F. (2021). Preclinical Bioavailability, Tissue Distribution, and Protein Binding Studies of Erinacine A, a Bioactive Compound from *Hericium Erinaceus* Mycelia Using Validated LC-MS/MS Method. Molecules.

[35] Roda E., De Luca F., Ratto D., Priori E.C., Savino E., Bottone M.G., Rossi P. (2023). Cognitive Healthy Aging in Mice: Boosting Memory by an Ergothioneine-Rich *Hericium Erinaceus* Primordium Extract. Biology.

[36] Hafliðadóttir S.H., Juhl C.B., Nielsen S.M., Henriksen M., Harris I.A., Bliddal H., Christensen R. (2021). Placebo Response and Effect in Randomized Clinical Trials: Meta-Research with Focus on Contextual Effects. Trials.

[37] Scholey A., Downey L.A., Ciorciari J., Pipingas A., Nolidin K., Finn M., Wines M., Catchlove S., Terrens A., Barlow E. (2012). Acute Neurocognitive Effects of Epigallocatechin Gallate (EGCG). Appetite.

[38] Vigna L., Morelli F., Agnelli G.M., Napolitano F., Ratto D., Occhinegro A., Di Iorio C., Savino E., Girometta C., Brandalise F. (2019). *Hericium Erinaceus* Improves Mood and Sleep Disorders in Patients Affected by Overweight or Obesity: Could Circulating Pro-BDNF and BDNF Be Potential Biomarkers?. Evid. Based Complement. Alternat. Med..

[39] Cryan J.F., O’Riordan K.J., Cowan C.S.M., Sandhu K.V., Bastiaanssen T.F.S., Boehme M., Codagnone M.G., Cussotto S., Fulling C., Golubeva A.V. (2019). The Microbiota-Gut-Brain Axis. Physiol. Rev..

[40] Fernandes A., Nair A., Kulkarni N., Todewale N., Jobby R. (2023). Exploring Mushroom Polysaccharides for the Development of Novel Prebiotics: A Review. Int. J. Med. Mushrooms.

[41] Higashi Y. (2019). Coffee and Endothelial Function: A Coffee Paradox?. Nutrients.

[42] Pardau M.D., Pereira A.S., Apostolides Z., Serem J.C., Bester M.J. (2017). Antioxidant and Anti-Inflammatory Properties of Ilex Guayusa Tea Preparations: A Comparison to *Camellia Sinensis* Teas. Food Funct..

